# Relationship between physical activity levels of Portuguese physical therapists and mental health during a COVID-19 pandemic: Being active is the key

**DOI:** 10.3389/fpubh.2022.986158

**Published:** 2022-11-01

**Authors:** Laura Cristina Almeida, Ana Grilo, Elisabete Carolino, Maria Teresa Tomás

**Affiliations:** ^1^Unidade de Cuidados Continuados Integrados de Portel—Unidade de Média Duração e Reabilitação e Unidade de Longa Duração e Manutenção, Santa Casa da Misericórdia de Portel, Portel, Portugal; ^2^ESTeSL – Escola Superior de Tecnologia da Saude de Lisboa, Instituto Politécnico de Lisboa, Lisboa, Portugal; ^3^H&TRC – Health and Technology Research Center, ESTeSL – Escola Superior de Tecnologia da Saúde, Instituto Politécnico de Lisboa, Lisboa, Portugal; ^4^CICPSI, Faculdade de Psicologia, Universidade de Lisboa, Alameda da Universidade, Lisboa, Portugal

**Keywords:** physical activity, COVID-19, physical therapists, mental health, pandemic

## Abstract

**Introduction:**

Physical activity is essential for a healthy life and quality of life, representing a fundamental role in individuals' physical and mental health. Concomitantly, the physical therapist, through the promotion of physical activity and exercise, can improve mental health, an essential factor in the current pandemic, triggering anxiety, fear, and depression crisis.

**Objective:**

To verify physical activity among Portuguese physical therapists and its association with mental health during pandemic times.

**Methods:**

An online questionnaire was applied through social media to all Portuguese physical therapists between October 21, 2021, and January 14, 2022. It contained general characterization questions of the sample, the IPAQ-SF questionnaire to assess physical activity levels, the Goldberg General Health Questionnaire (GHQ-28), which assesses the levels of mental health and the WHO Well-Being Index (WHO-5) to assess the subjective wellbeing.

**Results:**

The sample totaled 286 respondents (82% female), with a mean age of 33. Of the total answers, 82% practiced physical activity, 45% had moderate levels of physical activity, and 19% vigorous. Physical therapists in these categories had lower values in the GHQ-28 and higher in the WHO-5. Those with better mental health also showed better subjective wellbeing (*r* = −0.571, *p* = 0.000).

**Conclusion:**

The data obtained showed that physical therapists mostly have moderate and vigorous levels of physical activity and that physical activity positively influences individuals' mental health and wellbeing, which proved to be a key factor due to the pandemic situation.

## Introduction

Physical activity (PA) is defined by the World Health Organization (WHO) (1) as any movement performed by the musculoskeletal system which leads to energy expenditure (2). It plays a fundamental role in preventing and treating many non-communicable diseases, such as diabetes, heart, lung, and oncologic diseases, among others, and regarding functionality, mobility, anxiety, and depression (3).

Health professionals are held responsible for promoting PA in their clinical practice to reduce non-communicable diseases and improve individuals' quality of life, reducing physical inactivity and sedentary lifestyles, which have become a severe public health problem with high costs to society (4, 5).

Once confronted with the diseases triggered by physical inactivity, health professionals are in a favorable position to advise sedentary individuals to become more active and initiate behavioral changes regarding PA (6).

Physical therapists are qualified health professionals who prescribe PA to prevent or treat comorbidities and promote health (7). This status must be used to implement changes in individuals' lifestyles, promoting PA practice, and the physical therapists must be a practical example of this lifestyle (8).

The current COVID-19 pandemic, first reported in December 2019 in China (9), has spread rapidly around the world, and on January 30, 2020, the WHO deemed the coronavirus an international public health emergency (10). Pandemics brought confinement, which led to a drastic change in the lives and habits of the population, who had to change behaviors, commitments, lifestyle, and work from one moment to the next. This change also affected sports, physical activities, gyms, swimming pools, and physical therapy clinics, among others, with their closure (11).

However, the confinement proved to be a barrier to maintaining an active lifestyle, because outdoor health activities started to trigger worries and fears (11). This brought to the lives of individuals, disturbances in several ways, and particularly at the level of mental health (12).

According to WHO (2022) “Mental health is a state of mental wellbeing that enables people to cope with the stresses of life, realize their abilities, learn well and work well, and contribute to their community. It is an integral component of health and wellbeing that underpins our individual and collective abilities to make decisions, build relationships and shape the world we live in. Mental health is a basic human right, and it is crucial to personal, community and socio-economic development (13).” Wellbeing, on the other hand, is defined as “the state of being comfortable, healthy, or happy.” according to the Oxford English Dictionary (14).

The pandemic due to COVID-19, brought with it implications in several factors, and one of the main ones was the mental health implication, with an increase in stress in the short and long term. According to statistics, during the 1st year of the pandemic, anxiety and depressive disorders increased by about 25% (13).

In a study conducted in Italy during confinement, changes in PA during the pandemic, as well as its impact on mental health, were analyzed. The method used was the completion of an online questionnaire, in which PA energy expenditure during 1 week before quarantine and 1 week during quarantine was calculated, using an adapted version of the IPAQ and psychological wellbeing using the WHO-5. Authors found (15) that regular PA decreased significantly when analyzed before and during the pandemic (Mean: 2,429 vs. 1,577 MET.min/week, *p* < 0.0001), and this was found in all age groups and particularly in males. A significant positive correlation was also found between PA variation and general wellbeing (*r* = 0.07541, *p* = 0.0002), which suggested that the decrease in PA levels, led to a negative impact on the psychological health and wellbeing of the population. In summary, after analyzing the results it was found that maintaining regular PA was critical for physical and mental health during the COVID-19 pandemic (15).

Also, in the United States, a study (16) was done with 3,052 individuals *via* an online questionnaire on time spent in moderate and vigorous PA (separately), sitting, and using technology, pre, and post-COVID-19. They also questioned individuals on PA guidelines, stress, loneliness, positive mental health, social connectedness, and depressive and anxiety symptoms. Individuals were divided according to United States PA guidelines, ≥8 h/day sitting, or ≥8 h/day in front of a screen, pre-, and post-COVID-19. It was found that 62% of the participants were female, aged 18–24 years (16.6% of the sample), and older than 75 years (9.3%). According to participants' responses regarding PA, those who were active before COVID-19 reported decreased practice after COVID-19 [mean change: −32.3% (95% CI: −36.3%, −28.1%)], while those who were previously inactive reported no differences [+2.3% (−3.5%, +8.1%)]. In summary, improving/maintaining PA practice and limiting time in front of the screen may reduce negative effects on mental health in situations of drastic social change (16).

Canal-Rivero et al. (17), also studied the impact of COVID-19 and the possible gender differences in mental health status and suicide in health care professionals. They obtained relevant changes in the GHQ-28 analysis regarding the dimension that evaluates somatic symptoms, reinforcing the need to evaluate the wellbeing of professionals, particularly in stressful situations such as the COVID-19 pandemic. There were also differences between genders during the time they were followed up. Namely women presented more psychological distress at the beginning, but the differences tended to disappear over time. In summary, they state that a proper mental health assessment and recommendations about healthy lifestyle practices can lead to improved attitudes in stressful situations and reduce somatic symptoms (17).

The confinement proved to be a barrier to maintaining an active lifestyle because outdoor activities started to trigger worries and fears (11). This brought individuals disorders in several ways, particularly at the level of mental health (12). The positive effects of PA have been widely documented for its role in physical health and the promotion of mental health, representing an essential activity for physical and psychological balance, related to the quality of life and healthy aging (5, 18). According to the study by Dwyer et al. (19), regular PA showed several benefits in physical and mental health during the current pandemic if the physical distance was maintained.

PA is thus fundamental for mental health, an essential factor in social confinement/isolation, which can trigger anxiety attacks, fear, and depression, as well as in sleep disorders where it can be determined as a non-pharmacological therapy for insomnia. On the other hand, PA also strengthens the immune system and helps prevent and control non-communicable diseases, which are risk factors for COVID-19 infection (18, 20, 21).

Physical therapists were one of the health care professions that was most demanded during the COVID-19 pandemic and, to our knowledge, there are no studies that relate these variables in this professional group. The objective of this study was to analyze physical activity levels of Portuguese physical therapists and the association with their mental health in pandemic times.

## Methodology

### Study design and population

A quantitative, observational, analytical, cross-sectional study was designed using a self-completion questionnaire, which collected data in a single moment.

The population comprised Portuguese physical therapists in all clinical contexts and practices. According to the Portuguese Association of Physiotherapists (APFisio) (22), it is estimated that there are around 12,000 practicing physiotherapists in the country; consequently, it was a significant sample, considering the confidence level of 95%, with a margin of error of 5%, 385 questionnaires answered (*p* = 0.05). This is a non-random sample, auto-selection sampling type (23).

Inclusion criteria were being a physical therapist. The exclusion criteria were not accepting the Informed Consent form to continue filling out the online questionnaire.

### Instruments

Data were collected using an online questionnaire developed on the Google forms platform, and data were collected from October 21, 2021, to January 14, 2022. The questionnaire was disseminated through digital platforms, Facebook (different groups), and WhatsApp. It was requested to be disseminated through the various higher education institutions that teach the physiotherapy course in Portugal, as well as the physiotherapists' association APFisio, through its newsletter on 06/12/2021.

The questionnaire was composed of four parts: general characterization; the International Physical Activity Questionnaire (IPAQ-SF), the Goldberg's General Health Questionnaire (GHQ-28), and the WHO Well-Being Index (WHO-5).

#### Characterization data

Sample characterization questions were asked: age, gender, and professional experience (assessed by the number of years as a physical therapist and the number of hours worked per day).

#### Physical activity level assessment (IPAQ-SF)

The International Physical Activity Questionnaire (IPAQ-SF) was used to assess PA in its short form. It followed the classification indicated in several studies (24–28) and agreed with the official IPAQ classification protocol (www.ipaq.ki.se) (29), in which individuals are classified in three PA levels. These levels are calculated based on the duration (minutes), frequency per week, and MET intensity (MET.min/week); this value is obtained through a weighted approximation of the total PA performed during 1 week (27).

The official IPAQ scoring protocol suggests that individuals should be divided into three levels of PA:

- Vigorously Active Level (VA):
  (a) Three days minimum of vigorous PA and energy expenditure of 1,500 MET-min/week;
  (b) A minimum of 7 days of PA, comprising a combination of moderate PA, vigorous PA, and walking, achieving a minimum of 3,000 MET-min/week.
- Moderately Active Level (MA):
  (a) Three or more days of vigorous PA, with a minimum time of 20 min/day;
  (b) Five or more days of moderate PA, vigorous PA, or walking, reaching a minimum of 30 min/day;
  (c) Five or more days of PA per week (moderate PA, vigorous PA, walking, or the sum of PA) for a minimum of 600 MET-min/week.
- Insufficiently Active Level (IA):
  (a) Participants whose energy expenditure does not reach PA values can be included in any previous categories.

We prioritized this questionnaire due to her short form and because it is less expensive (30). After all, it is feasible to administer online (shorter). Extended versions seem to overestimate PA levels (27). Also, it has been developed and tested, particularly to assess PA levels in the adult population, namely in the 15–69 age group (31).

#### Mental health evaluation (GHQ-28)

The Goldberg's General Health Questionnaire (GHQ-28) was used to evaluate Mental Health. It presents 28 questions divided into four subscales that assess four dimensions: somatic symptoms subscale (questions 1–7), anxiety and insomnia subscale (questions 8–14), social dysfunction subscale (questions 15–21), and major depression subscale (questions 22–28) (32–35).

The questionnaire created by Goldberg and Hiller in 1979 (36) uses a Lickert-type scale. The total score is the sum of the scores of the four dimensions or subscales. It is also possible to obtain the score for each subscale. In each question, the score ranges from “0” to “3,” and per dimension, it ranges from “0” to “21.” Therefore, the questionnaire's total score range between “0” and “84,” where higher values represent poor mental health (33, 36).

Several studies refer to different cut-off points for each subscale, 4/5 (37), or 5/6 (23), and in the total scale value, they use the value of 22 (32) or 23/24 (38, 39).

It is a self-administered questionnaire that allows the assessment of mental health or psychological wellbeing among the general population in non-psychiatric settings, such as public health and primary health care (38).

It is a clinical assessment method that allows quick results to identify possible psychiatric cases to be subsequently diagnosed (40).

In this study, the Portuguese version (38) of the Goldberg General Health Questionnaire (GHQ-28) was used.

#### Assessment of subjective wellbeing (WHO-5)

WHO Well-Being Index (five) (WHO-5) (41), 1998 version, a short self-completion scale, was used to assess subjective wellbeing.

This index is widely used in research worldwide, its first publication dating from 1998, and has been translated into more than 30 languages (42). It was initially created to evaluate subjective wellbeing, but there is also evidence of it in the diagnosis of depression (43).

WHO-5 consists of five positively worded questions that ask which way is closest to how the individual has felt over the past 2 weeks. The values are scored on a six-point Likert-type scale ranging from 0 (Never) to 5 (All the time). Questions scores can be modified on a scale from 0 to 100, where higher scores indicate a better level of wellbeing (41, 44, 45).

### Ethical procedures

The online questionnaire was anonymized to safeguard the privacy of the participants. It included a brief description of the study, its objective, and the informed consent, following the Helsinki Declaration (dated 1964).

This study was submitted to the Ethics Committee (EC) and the Scientific-Technical Council of the Escola Superior de Tecnologia da Saúde de Lisboa (ESTeSL)—IPL, obtaining a favorable opinion (CE-ESTeSL-N°30-2021 on 08/10/2021).

### Statistical analysis

Results were analyzed in the Statistical Package for Social Sciences (IBM SPSS Statistics), version 26.

The results were considered significant at a 5% significance level. To characterize the sample, frequency analysis (*n*, %) for qualitative data was used, and mean and standard deviation for quantitative data was employed. Data normality was verified using the Kolmogorov-Smirnov test.

For the comparison of *k* > 2 independent groups, the Kruskal-Wallis test was used since the normality assumption was not verified.

## Results

### Characterization of the sample

The sample was made up of 289 questionnaires (*n* = 289). Three participants answered, “I do not want to participate in this study, or I am not a physical therapist,” so they ended the questionnaire, therefore, they were excluded from the study. Thus, the final sample consisted of 286 participants. Of the total respondents, 82.2% were female, and 17.8% were male, ranging from 21 to 67 years (33 ± 10 years). The age of the participants was defined as young adults (≥40 years), adults (41–60 years), and over 60 years (41) (Table 1).

**Table 1:** Sample characterization.

		***N* (%)**	**Mean ±SD (range)**
Gender	Female	235 (82.2)	
	Male	51 (17.8)	
Age (years)		285	33.1 ± 9.9 (21–67)
Age group	Under 40 years	230 (80.7)	
	Between 41 and 60 years	54 (18.9)	
	Over 61 years	1 (0.4)	

N, Frequency; %, Percentage; SD, Standard Deviation.

In relation to the population of physiotherapists registered in the physiotherapists' order, the average age is 34.4+-9.1 years, with 26.1% men and 73.9% women. Regarding these values, statistically significant differences were detected in the study sample (*p*'s < 0.05). In the sample, on average, ages are lower and there is a higher percentage of women. However, despite the differences, the trend of the sample is the same as the population.

### Physical activity levels

Of the 286 individuals who answered the questionnaire, 235 (82.2%) indicated they practiced PA, while 51 (17.8%) did not practice any PA (Table 2). Regarding the practice of vigorous PA, 54.2% practiced some days a week, 42.7% indicated that they did not practice, and 3.1% indicated that they did not know or were not sure. Regarding moderate PA, 73.8% answered that they practiced some days a week, 22.4% did not practice, and 3.8% did not know or were not sure. When asked about the days those physical therapists spend walking for more than 10 min, 216 (75.5%) individuals indicated that they do it a few days a week, and 53 (18.5%) do not walk daily for more than 10 min, and 5.9% do not know or are not sure. Of these, only 201 indicated the number of days they performed walking (4 ± 2 days).

**Table 2:** PA habits and levels of physical therapists per week in pandemic time (IPAQ-SF).

	***N***	**%**	**Mean ±SD (range)**
Practicing PA	235	82.2	
Does not practice PA	51	17.8	
Insufficiently Active	104	36.4	
Moderately active	129	45.1	
Vigorously active	53	18.5	
Minutes per day sitting	242		221 ± 175 (15–1200)

N, Frequency; %, Percentage; SD, Standard Deviation; PA, Physical Activity; IPAQ-SF, International Physical Activity Questionnaire-Short Form.

Regarding the PA levels of the Portuguese physical therapists, it was found that most (45.1%) of the participants had moderate levels of PA, 36.4% were IA (Insufficiently active), and 18.5% were VA (Vigorously active) (Table 2).

It was also possible to analyze from the IPAQ-SF questionnaire the minutes per day spent in sedentary behavior, namely sitting, presenting a range value between 15 and 1,200-min sitting (221 ± 175) (Table 2).

### Mental health

The mental health of physical therapists was assessed through the GHQ-28, obtaining the following data: In the subscale of Somatic Symptoms, a mean of 4.87 ± 3 was acquired; regarding Anxiety and Insomnia, a mean of 6.53 ± 5 was obtained, and in the subscale of Social Dysfunction 8.64 ± 3, and the Severe Depression they presented the lowest value of 1.62 ± 3. To these answers corresponded the total (mean) value of the scale of 22 ± 9 points (Table 3).

**Table 3:** Results of the GHQ-28 and WHO-5 questionnaires (*n* = 286).

	**Mean ±SD**
**GHQ-28 results**	
Somatic symptoms	4.9 ± 3.3
Anxiety and insomnia	6.5 ± 4.8
Social dysfunction	8.6 ± 3.2
Major depression	1.6 ± 3.0
*Total score*	21.7 ± 9.1
**WHO-5 results**	
Percentage of WHO-5	59.0 ± 18.6

SD, Standard Deviation; GHQ-28, General Health Questionnaire-28; WHO-5, WHO Well-Being Index (five).

Regarding the mental health of Portuguese physical therapists, the following GHQ-28 results were obtained (Table 3).

### Wellbeing

In the analysis of wellbeing through the WHO-5, most participants found results between 51 and 75% (59 ± 19), thus verifying a medium/high sense of wellbeing since 0 represents the worst and 100 represents the best sense of wellbeing (Table 3).

### Association between levels of physical activity and mental health

The association between PA levels and mental health was analyzed through IPAQ-SF and GHQ-28 results, and the following was found.

**Figure 1:**
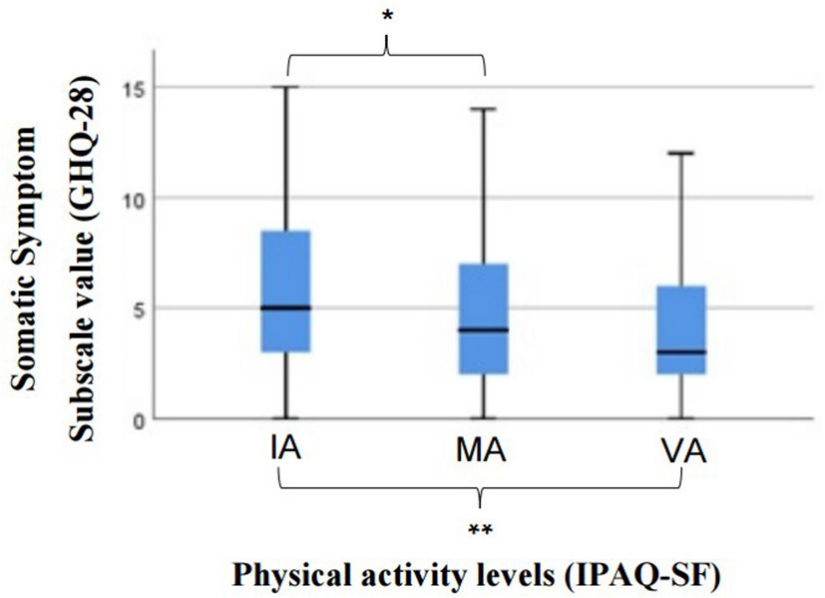
Results of the Kruskal-wallis test for the comparison of somatic symptoms among the physical activity levels. GHQ-28, General Health Questionnaire-28; AI, Insufficiently Active; MA, Moderately Active; VA, Vigorously Active; PA, Physical Activity; IPAQ-SF, International Physical Activity Questionnaire; *Significant differences between the category IA and MA individuals (*p* = 0.012); **Significant differences between IA and VA individuals (*p* = 0.005).

Regarding Somatic Symptoms (GHQ-28) differed between at least two groups of the levels of PA [$x_{\text{kw}}^{2}$(2) = 9.889, *p* = 0.007]. The multiple comparisons test regarding PA levels found significant differences between the category IA and MA individuals (*p* = 0.012) and between IA and VA individuals (*p* = 0.005). Individuals in the IA category showed higher values on the Somatic Symptoms subscale than individuals practicing PA of moderate or vigorous intensity (Figure 1).

**Figure 2:**
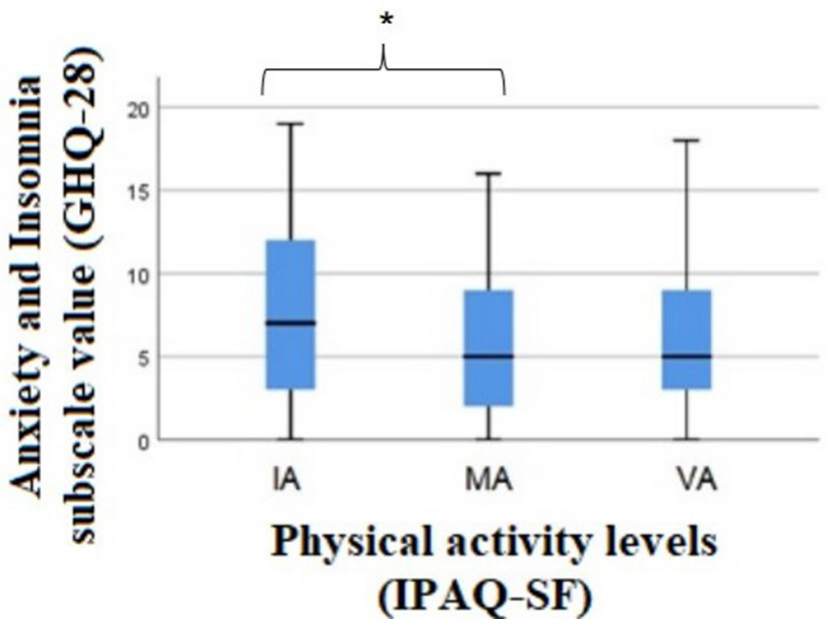
Results of the Kruskal-wallis test for the comparison of anxiety and insomnia between the levels of physical activity. GHQ-28, General Health Questionnaire-28; AI, Insufficiently Active; MA, Moderately Active; VA, Vigorously Active; PA, Physical Activity; IPAQ-SF, International Physical Activity Questionnaire; *Significant differences between the category IA and MA individuals (*p* = 0.005).

In the Anxiety and Insomnia subscale, PA levels differed in at least one group [$x_{\text{kw}}^{2}$(2) = 7.773, *p* = 0.021]. The multiple comparisons concluded that there are significant differences when comparing individuals with insufficient levels of PA (IA) and MA (*p* = 0.005). In this subscale, it was observed that MA individuals presented the best results (lower values) (Figure 2), i.e., they presented minor anxiety and insomnia symptoms.

In the subscale of Social Dysfunction [$x_{\text{kw}}^{2}$(2) = 1.314, *p* = 0.518] and Severe Depression [$x_{\text{kw}}^{2}$(2) = 3.823, *p* = 0.148], there were no significant differences between the three levels of PA.

**Figure 3:**
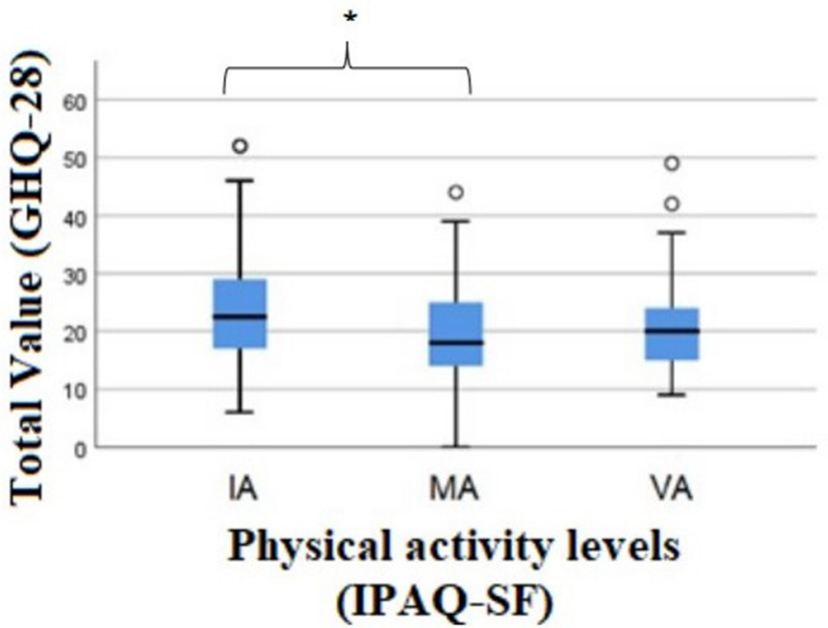
Results of the Kruskal-wallis test for the comparison of total value (GHQ-28) between the levels of physical activity. GHQ-28, General Health Questionnaire-28; AI, Insufficiently Active; MA, Moderately Active; VA, Vigorously Active; PA, Physical Activity; IPAQ-SF, International Physical Activity Questionnaire; *Significant differences between the category IA and MA individuals (*p* = 0.007).

Regarding the total score of the GHQ-28 scale, there were differences between groups [$x_{\text{kw}}^{2}$(2) = 9.408, *p* = 0.009]. When the test of multiple comparisons was used, there was a significant difference between IA and MA individuals (*p* = 0.007), in which it was verified that IA individuals presented higher values in the total score of the scale than MA individuals (Figure 3).

According to the authors of the scale (31), higher values on the GHQ-28 correspond to lower levels of mental health, and it is verified that individuals who practice less PA have a worse value of mental health than those who practice MA, according to the total value.

### Association between subjective wellbeing and physical activity levels

**Figure 4:**
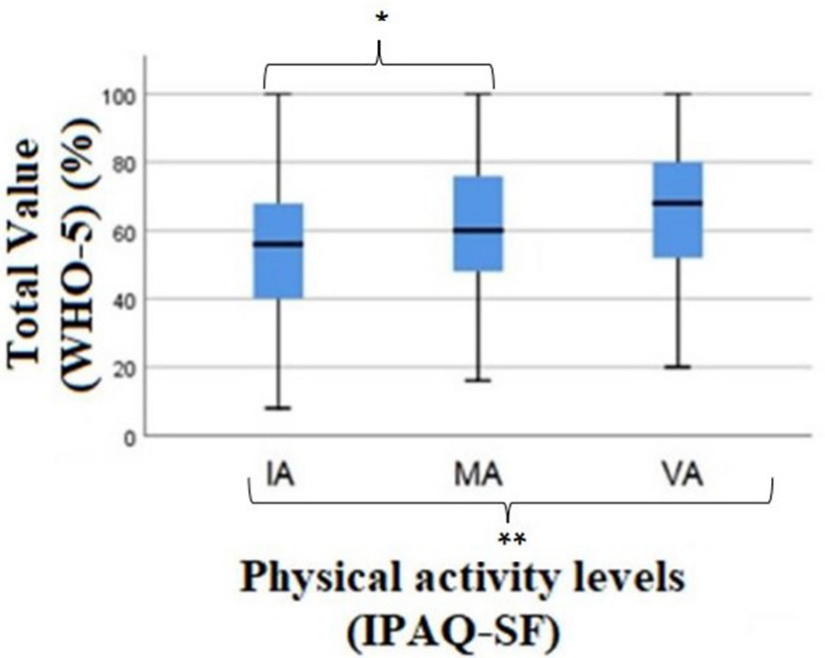
Results of the Kruskal-wallis test for the comparison of total value (WHO-5) (%) between the levels of physical activity. WHO-5, WHO Well-Being Index (five); IA, Insufficiently Active; MA, Moderately Active; VA, Vigorously Active; PA, Physical Activity; IPAQ-SF, International Physical Activity Questionnaire; *Significant differences between the category IA and MA individuals (*p* = 0.013); **Significant differences between IA and VA individuals (*p* = 0.000).

Analysis of the WHO-5(%) questionnaire and PA levels revealed differences between at least one of the groups [$x_{\text{kw}}^{2}$(2) = 16.645, *p* < 0.001]. From the multiple comparisons, differences were verified between the participants who are in category IA and MA (*p* = 0.013), analyzing that the individuals who are in category IA presented lower values in the questionnaire, therefore a lower sense of wellbeing, the same was verified between IA and VA (*p* < 0.001) (Figure 4).

## Discussion

This study was developed to verify Portuguese physical therapists' physical activity levels (PA) and their association with mental health during the pandemic.

A sample of 286 physical therapists was obtained, of which the majority (82.2%) were female and 17.8% male. The study by Kgokong et al. (46), with identical objectives to the present study, was only carried out with physical therapy students. The proportion between females and males was identical (83% female).

Regarding the age of the participants, the age group that presented the fewest answers to the questionnaire was the group over 61 years old, with only one respondent. Younger physical therapists adhered more significantly, with 80.7% of participants aged 40 years or younger. This leads to considering that younger individuals have a greater propensity and willingness to answer online questionnaires or higher use of digital platforms.

In our sample, 82.2% practice PA, while 17.8% reported they do not practice any PA, as well as, in the study by Black et al. (47), in which 80.8% of the participants reported they practice regular PA.

When analyzing the study by Chevan and Haskvitz (48), physical therapist assistants and students seem to follow the ACSM recommendations for PA compared to the rest of the general population and other health professionals. That means the professional is aware and motivated about the importance of regular PA and its benefits. Physical therapists should be a practical example of what they teach and promote, encouraging the population to acquire healthier behaviors through regular PA practice (47, 49).

By calculating the PA levels of the physical therapists in the present study, based on the duration (minutes), frequency (per week), and MET intensity (MET.min/week) (27), it was found that the PTs are mostly moderately active (MA) and vigorously active (VA). Also, in the study by Antunes et al. (50), the practice of PA during the COVID-19 pandemic in Portugal was analyzed, and most participants practiced regular PA. The IPAQ results showed moderate and vigorous levels of PA.

When we analyzed the values obtained by the PTs in our study, we found that the scale from somatic symptoms to major depression and the total value of the scale was below the cutoff point, which would be expected since we were analyzing data from a population with no diagnosis of mental illness (38).

When comparing the results of the present study with the values obtained by Ribeiro and Amaro (23), who assessed the mental health of social workers in Portugal, it was found that the present sample obtainable lower values in all subscales except the social dysfunction subscale. However, comparing the present study with the study of Sá (51), which analyzed 416 Portuguese nurses' mental health, the present physical therapists' sample showed all subscales with higher values, except for somatization. In this subscale, the physical therapists presented a mean of 4.87 and the nurses a mean of 6.047 (51).

These results suggest that physical therapists have a lower incidence of symptoms compatible with emotional disorders than social workers (23) and a higher incidence than nurses in Sá study (51). However, regarding the results obtained in the present study, it is essential to consider that the data was collected during the COVID-19 pandemic, a factor that has triggered negative psychological consequences, increasing anxiety levels, stress, fear, and even depression (50). During the pandemic, one of the main protection measures was quarantine and physical distance to mitigate the spread of the virus. However, these measures enhance adverse effects at the psychosocial level (52), which may have influenced GHQ-28 values obtained in the present research.

PA is essential not only for physical health but also for mental health (50). Thus, it was necessary to verify that PA levels of PTs influence their mental health in a difficult situation such as a pandemic, relating to the results obtained in the IPAQ-SF with the results obtained in the GHQ-28.

It has been found that individuals with lower PA values are more likely to be emotionally disturbed concerning somatization (23).

When we analyze the anxiety and insomnia scale, MA subjects have lower values on this subscale than IA subjects, showing less anxiety and insomnia symptoms.

When looking at the total scale score, individuals who are MA have lower scores on the scale than AI individuals. Furthermore, studies indicate (23, 37) higher values on the GHQ-28 correspond to lower mental health levels. Therefore, in our study, IA physical therapists individuals had lower mental health scores than those who practiced PA (namely, MA).

Analysis of subjective wellbeing (WHO-5) and the levels of PA (IPAQ-SF) results confirmed. That is, physical therapists who were IA had lower feelings of subjective wellbeing when compared to physical therapists who were MA and VA. Also, in a recent study (15) that analyzed the impact of PA on psychological health during the pandemic, there was a positive correlation between PA and wellbeing in which decreased levels of PA correlated with decreased feelings of wellbeing (*r* = 0.07541, *p* = 0.0002).

## Limitations

It is believed that the main limitation of this study will be our sample size since, to be representative of the population in question (about 12,000 Physical therapists), we would need at least 385 individuals.

Also, the type of data collection may generate biases since there is the possibility that the people who make up the sample are selected by convenience and not randomly. Furthermore, to justify the lower number of individuals in older age groups, we believe that older individuals are more reluctant to answer this questionnaire or are fewer users of existing digital platforms.

Our study, had only the purpose of analyze levels of PA in Portuguese physical therapists and related mental health and wellbeing in a pandemic context but a limitation founded was the lack of covariates (e.g., context in which physical activity is practiced—at home/outdoor, alone or with others, levels of training, history of physical activity, reason not to practice physical activity, etc.) as a threat to internal validity. This should be taken in account in future studies.

The results obtained may thus serve as a basis for future studies, which should be carried out with a more significant sample of the population, and perhaps through another type of data collection (random) to obtain more significant values and a more heterogeneous sample.

## Conclusion

To our knowledge, this is the first study that evaluates the physical activity levels of physical therapists in Portugal and especially relates this data to mental health during a time that involved so many changes in how they live and work.

The present study show that PA positively influences physical therapists' mental health, as well as their feeling and wellbeing, which are critical factors given to the pandemic situation. Moreover, most physical therapists seem to be aware of regular PA practice, although there is still space for improvement since almost 40% of insufficiently active physical therapists are yet absent.

Since PA influences mental health, the physical therapist as a health professional needs to have good levels of mental health to provide better care to their users. According to the results and considering that most physical therapists have moderate and vigorous levels of PA, the profession is in a beneficial position regarding mental health and wellbeing.

Mental health and physical inactivity are two challenges for public health, mainly due to its social and economic determinants, which have worsened in the current pandemic of COVID-19. Thus, it would be relevant to review the physical therapist's performance as health promotors, so that through the prescription and counseling of PA, it is possible to reduce the incidence of disease and improve the physical and mental health of the general population.

## Data availability statement

The raw data supporting the conclusions in this paper will be made available by the authors without undue reservation.

## Author contributions

LA designed the study, designed the questionnaire, analyzed and interpreted the results, and wrote the manuscript. EC helped with statistical analysis of results. MT and AG revised the study and all the manuscript and with LA approved the final version. All authors contributed to the article and approved the submitted version.

## Funding

This project was supported by FCT/MCTES (UIDB/05608/2020 and UIDP/05608/2020).

## Conflict of interest

The authors declare that the research was conducted in the absence of any commercial or financial relationships that could be construed as a potential conflict of interest.

## Publisher's note

All claims expressed in this article are solely those of the authors and do not necessarily represent those of their affiliated organizations, or those of the publisher, the editors and the reviewers. Any product that may be evaluated in this article, or claim that may be made by its manufacturer, is not guaranteed or endorsed by the publisher.

## Abbreviations

APFisio: Portuguese Association of Physiotherapists
COVID-19: Coronavirus Disease 2019
GHQ-28: General Health Questionnaire - 28 items
IA: Insufficiently active
MA: Moderately active
VA: Vigorously active
IPAQ-SF: International Physical Activity Questionnaire
MET: Task Metabolic Equivalent
PA: Physical activity
WHO: World Health Organization
WHO 5: WHO Well-Being Index.
